# Adjunctive screw fixation and screw corridors for spiral tibial fractures involving the distal tibial articular surface: a retrospective case series

**DOI:** 10.3389/fsurg.2026.1944619

**Published:** 2026-10-22

**Authors:** Xianjie Ai, Zhimeng Wang, Yuhang Liu, Chengcheng Zhang, Hongfei Qi, Shuai Ji, Bing Du, Zhong Li, Ming Li, Bo Wu

**Affiliations:** 1 Honghui Hospital, Xi'an Jiaotong University, Xi'an, Shaanxi, China; 2 Medical College of Yan'an University, Yan'an, Shaanxi, China

**Keywords:** adjunctive screw fixation, computed tomography, distal tibial articular surface, intramedullary nail, screw corridor, spiral tibial fracture

## Abstract

**Objective:**

Building on a CT-based regional evaluation framework developed in our previous morphological study, this study retrospectively summarized the adjunctive screw corridors actually used and their fixation targets in spiral tibial fractures involving the distal tibial articular surface that were treated with intramedullary nailing combined with adjunctive screw fixation, and used representative intraoperative and postoperative images to illustrate the relationship between the adjunctive screws and the intramedullary nail.

**Methods:**

This was a single-center retrospective case series of patients with spiral tibial fractures involving the distal tibial articular surface who underwent tibial intramedullary nailing combined with adjunctive screw fixation at the Department of Lower Limb Trauma, Honghui Hospital, Xi'an Jiaotong University, between May 2020 and May 2025. During the study period, 110 patients were treated with this combined fixation; 98 patients with a follow-up duration of at least 12 months and complete clinical and imaging records were included in the analysis, and 12 patients were excluded because of insufficient follow-up or incomplete imaging records. Two observers independently assessed the involvement of the posterior malleolar/Volkmann region, the anterior malleolar/Chaput region, and the medial malleolar region on preoperative CT, and Cohen's kappa was calculated from the original independent assessments obtained before consensus discussion. The four types of adjunctive screw corridors actually used were retrospectively summarized from operative records and postoperative imaging. All patients underwent adjunctive screw fixation before reaming and intramedullary nail insertion. Corridor use was summarized using descriptive statistics, and perioperative variables, time to full weight bearing, the American Orthopaedic Foot & Ankle Society (AOFAS) ankle-hindfoot score, the visual analog scale (VAS) pain score, the difference in ankle dorsiflexion angle between the affected and contralateral sides, and related complications were recorded.

**Results:**

A total of 98 patients were included, comprising 62 men and 36 women, with a mean age of 45.67 ± 13.61 years. The posterior malleolar/Volkmann region, the anterior malleolar/Chaput region, and the medial malleolar region were involved in 76 (77.6%), 40 (40.8%), and 23 (23.5%) patients, respectively; single-region, two-region, and three-region involvement was present in 64, 27, and 7 patients. Interobserver agreement for the individual regions and for their combinations ranged from 95.9% to 98.0%, with kappa values of 0.924–0.957. The tibiofibular incisura, anterior cortical, posterior cortical, and medial malleolar corridors were used in 93, 8, 7, and 6 patients, respectively; 87 patients received a single corridor and 11 received multiple corridors. The number of adjunctive screws ranged from 1 to 4, with a mean of 1.23 ± 0.62. Mean operative time was 73.45 ± 7.88 min, mean intraoperative blood loss was 67.56 ± 10.46 mL, and mean follow-up was 16.13 ± 2.20 months. Ninety-seven patients were included in the analyses of time to full weight bearing and final functional outcomes. Mean time to full weight bearing was 64.81 ± 16.91 days; at the final follow-up, the mean AOFAS ankle-hindfoot score was 91.67 ± 3.13, the mean VAS pain score was 1.36 ± 0.50, and the mean difference in ankle dorsiflexion angle between sides was 6.06 ± 3.27 degrees. Two patients developed delayed healing of the anterior ankle incision used for distal locking screws, which healed after dressing changes, and one patient developed an infection at the tibial shaft fracture site and was treated with implant removal and antibiotic-loaded bone cement. Among the 98 patients with complete follow-up, no obvious articular redisplacement or fixation failure was observed on the available follow-up radiographs.

**Conclusion:**

This retrospective case series summarized the four types of adjunctive screw corridors actually used when spiral tibial fractures involving the distal tibial articular surface were treated with intramedullary nailing combined with adjunctive screw fixation, and described the fixation target of each corridor. Representative images are used to illustrate, but do not systematically analyze, the relationship between the adjunctive screws and the intramedullary nail. These results provide descriptive information on the adjunctive fixation technique used in this cohort, and their value for guiding preoperative planning and surgical decision-making requires further validation.

## Introduction

Spiral tibial fractures are usually caused by rotational forces. When the main fracture is located in the middle-distal segment or the distal tibia, the fracture line may extend distally and may be accompanied by injury to the distal tibial articular surface and the peri-ankle osseous structures (1–3). Some articular fracture lines are subtle and minimally displaced, and conventional radiographs may underestimate their occurrence and true extent (4, 5). CT can further identify associated fractures involving the posterior malleolar, anterior malleolar, and medial malleolar regions, and some patients have simultaneous involvement of multiple regions (6). In this article, “occult articular fracture” refers to a distal tibial articular fracture that is not clearly identified on conventional radiographs but is confirmed by CT; the term describes imaging detectability and is not equivalent to a non-displaced fracture.

Intramedullary nailing can restore and maintain tibial length, alignment, and rotational stability, and it has been widely used in the treatment of tibial shaft fractures and selected distal tibial fractures (7, 8). However, when the fracture also involves the distal tibial articular surface, the intramedullary nail mainly stabilizes the main fracture component and cannot necessarily control independent articular fragments. Previous clinical and morphological studies have suggested that such associated injuries should be identified preoperatively and managed according to the specific fracture morphology (9–12). For articular fragments that have not yet been stably fixed, secondary displacement may occur during reaming and nail insertion; previous studies on the sequence of fixation have also provided some clinical basis for addressing articular fractures before intramedullary nail insertion (13).

Previous studies have reported the use of intramedullary nailing combined with adjunctive screws for tibial shaft or distal tibial fractures with simple articular extension, with a focus mainly on fracture healing, limb alignment, and clinical outcomes (14–19). In contrast, rather specific technical information is still limited regarding how the adjunctive screws actually run, which fragments they mainly connect, and how their spatial relationship with the distal end of the intramedullary nail and the distal locking screws is managed.

Our previous CT-based morphological study described the regional distribution and fracture-line course of spiral tibial fractures involving the distal tibial articular surface, and showed that such injuries may involve the posterior malleolar/Volkmann region, the anterior malleolar/Chaput region, and the medial malleolar region, as well as different combinations of these regions (20). Building on that work, the present study combined operative records and postoperative imaging to retrospectively summarize the actual application of four types of adjunctive screw corridors in patients treated with intramedullary nailing combined with adjunctive screw fixation, focusing on their fixation targets and illustrating, with representative images, the relationship between the adjunctive screws and the intramedullary nail. This study does not re-verify the previously reported distribution of articular involvement, nor does it aim to establish a one-to-one correspondence between a given fracture morphology and a specific screw corridor, or a corridor-selection algorithm.

## Materials and methods

### Study design and patient source

This was a single-center retrospective case series. Patients treated in the Department of Lower Limb Trauma, Honghui Hospital, Xi'an Jiaotong University, between May 2020 and May 2025 were screened through the hospital case system. The study population comprised patients with spiral tibial fractures involving the distal tibial articular surface who were treated with tibial intramedullary nailing combined with adjunctive screw fixation. During the study period, 110 patients were treated with this combined fixation. Ninety-eight of these patients had a follow-up duration of at least 12 months with complete clinical and imaging records and were included in the analysis. The remaining 12 patients had a follow-up duration shorter than 12 months or incomplete imaging records and were excluded. The four corridors summarized in this article were not a prespecified condition for case retrieval or inclusion. The patient selection process is shown in Supplementary Figure S1.

Of the 98 patients, 89 had been included in our previous CT morphological study of 160 patients, and 9 were new patients treated between January and May 2025. The previous study included patients treated between May 2020 and December 2024, and the 89 overlapping patients used the same preoperative CT dataset obtained at the time of injury in both studies (20). Unlike the previous study, the present study focused on the adjunctive fixation actually used, based on operative records and postoperative imaging; the morphological data served only to describe the injury background of this cohort and were not used as an independent cohort to re-verify previously reported morphological patterns. The fracture-line distribution map and heat map followed the mapping method of the previous study but were both redrawn using the data of the present 98 patients; images from the previous study were not directly reused.

This study was approved by the Ethics Committee of Honghui Hospital, Xi'an Jiaotong University (approval no. 202410008). It used existing clinical data, and all patient data were anonymized. The ethics committee waived the requirement for informed consent for this retrospective study.

### Eligibility criteria

The inclusion criteria were: (1) age ≥ 18 years; (2) a closed spiral tibial fracture with the main fracture located in the tibial shaft or the distal tibia; (3) preoperative CT showing involvement of the distal tibial articular surface, including extension of the fracture line to the articular surface or an occult articular fracture; (4) preoperative CT of sufficient quality for three-dimensional reconstruction and assessment of the involved regions; (5) treatment with tibial intramedullary nailing combined with adjunctive screw fixation; and (6) a follow-up duration of at least 12 months.

The exclusion criteria were: (1) pathological fracture; (2) open fracture; (3) a typical pilon fracture, particularly an AO/OTA 43B or 43C high-energy compressive articular fracture; (4) a history of ipsilateral ankle deformity or severe trauma; (5) severe comminution that prevented accurate assessment of the fracture-line course and the involved articular regions; and (6) incomplete clinical data or imaging follow-up.

Spiral fractures were identified mainly by the spiral course of the fracture line along the tibia on imaging, in combination with the injury mechanism and the AO/OTA classification. The AO/OTA codes in Table 1 describe the main tibial fracture component, whereas the associated distal articular injury was recorded separately on CT according to the involved regions and their combinations; this recording approach follows our previous morphological study (20). Specifically, the 43A1 code describes the main distal tibial fracture component in 13 patients and does not indicate that the overall injury in these patients was extra-articular.

**Table 1:** Demographic and fracture characteristics of the patients.

Variable	Value
Number of patients	98
Age, years	45.67 ± 13.61
Male/female	62/36
Left/right side	42/56
AO/OTA type 42A1	54
AO/OTA type 42B1	23
AO/OTA type 42C1	8
AO/OTA type 43A1	13
With fibular fracture	95
Without fibular fracture	3
Fibular fracture, no direct fixation	55
Fibular fracture, plate fixation	34
Fibular fracture, intramedullary wire fixation	6
Fibular fracture location: distal fibula	23
Fibular fracture location: lower shaft	13
Fibular fracture location: middle shaft	15
Fibular fracture location: upper shaft	17
Fibular fracture location: proximal fibula (head and neck)	21
Fibular fracture location: proximal fibula + middle shaft	2
Fibular fracture location: upper shaft + distal fibula	4
Slip and fall	59
Electric bicycle fall	16
Fall down stairs	8
Road traffic injury	8
Bicycle fall	4
Crush injury by a falling heavy object	2
Fall from height	1

Data are presented as mean ± standard deviation or number of patients. The AO/OTA code describes the main tibial fracture component; associated involvement of the distal tibial articular surface was recorded separately on CT. Fibular fracture location and management are reported for the 95 patients with an associated fibular fracture; patients with involvement of two fibular levels are listed separately, and the location categories sum to 95. AO/OTA, Arbeitsgemeinschaft fur Osteosynthesefragen/Orthopaedic Trauma Association.

**Table 2a:** Distribution patterns of distal tibial articular involvement.

Involved region	n	Percentage
Posterior malleolar/Volkmann region	76	77.6%
Anterior malleolar/Chaput region	40	40.8%
Medial malleolar region	23	23.5%

One patient could have involvement of multiple regions; therefore, the sum of percentages may exceed 100%.

### CT regional assessment and imaging mapping

All patients underwent preoperative full-length tibial radiography and CT. Axial, coronal, and sagittal images and three-dimensional reconstructions were used to record involvement of the distal tibial articular surface according to the anatomical regional assessment method used in our previous study (20) (Figure 1). The regional boundaries were defined by anatomical landmarks, and no additional quantitative geometric boundaries were set.

**Figure 1:**
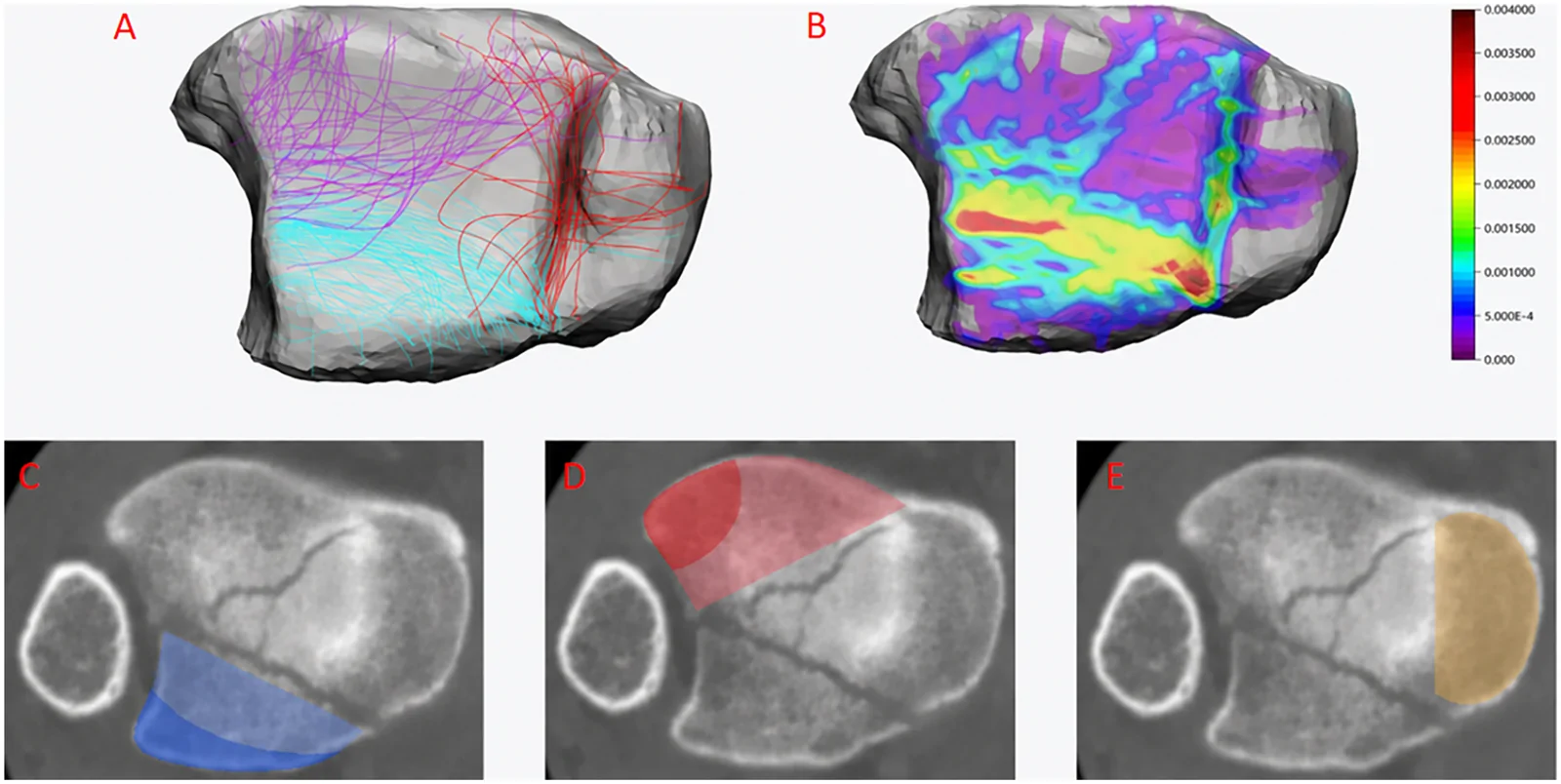
Regional evaluation framework for distal tibial articular surface involvement. **(A)** Three-dimensional fracture-line distribution map. **(B)** Fracture-line density heat map. Panels A and B followed the fracture-line mapping method of our previous CT morphological study but were newly generated for the present study from the data of the 98 patients included here; images from the previous study were not reused. **(C)** Posterior malleolar/Volkmann region (blue). **(D)** Anterior malleolar/Chaput region (red). **(E)** Medial malleolar region (yellow). The shaded areas indicate the anatomical regions recorded as involved; regional assignment was based on anatomical landmarks rather than on quantified geometric boundaries. This framework was used only for retrospective imaging classification and analysis of involvement patterns in this study; it was neither a prespecified intraoperative decision-making tool nor an independently validated fracture classification system.

The posterior malleolar/Volkmann region was defined as the region of the posterior malleolus or the Volkmann tubercle; the anterior malleolar/Chaput region as the anterior articular surface of the distal tibia or the region of the Chaput tubercle; and the medial malleolar region as the medial malleolar articular surface or the junction between the medial malleolar prominence and the articular surface. For fracture lines located at the junction between regions, multiplanar CT and three-dimensional reconstruction were used to determine the anatomical structures actually involved by the fracture line and the corresponding fragment. When multiple regions were involved, each involved region and its combination were recorded separately, and the fracture line was not forced into a single region. Small fragments were likewise recorded according to the articular surface actually involved, and involvement of a region was not disregarded because of small fragment size.

This regional framework was used only for retrospective imaging description in this study. It is not a new fracture classification system, nor was it a prespecified surgical decision-making tool during the study period. Recording the involvement of a region does not mean that every fracture line or every small fragment in that region required fixation with a separate screw.

### Interobserver agreement

Two surgeons with more than 5 years of clinical experience in orthopedic trauma independently reviewed all preoperative CT scans and recorded the involvement of the posterior malleolar/Volkmann, anterior malleolar/Chaput, and medial malleolar regions and the combinations of these regions; neither observer was aware of the other's assessment during independent interpretation. Cohen's kappa, the agreement rate, and the 95% confidence interval of kappa were all calculated from the original independent assessments of the two observers obtained before the consensus discussion.

For cases with discordant assessments, the two observers jointly reviewed the multiplanar CT and three-dimensional reconstruction images; if disagreement persisted, a senior orthopedic trauma surgeon with more than 10 years of clinical experience participated in the discussion and determined the final classification. The final classification obtained after review was used for the subsequent descriptive analysis of involved regions and corridor use, and did not replace the original independent assessments used for the interobserver agreement analysis.

### Adjunctive fixation principles and surgical decision-making

All procedures in this cohort were performed by the same surgical team, and the decision to use adjunctive fixation and the specific fixation plan were made by the same senior chief physician. The shared principle in this cohort was that adjunctive screw fixation was performed once preoperative CT confirmed a fracture involving the distal tibial articular surface, and no preset numerical threshold for articular step-off, fracture gap, or fragment size was used as an indication for fixation. For articular fragments that were already displaced, the fragment was reduced and then connected to the main fracture component; for fragments without displacement or with minimal displacement, adjunctive fixation was mainly intended to reduce the risk of secondary displacement during reaming and intramedullary nail insertion. The “main fracture component” refers to the fragment that was subsequently connected to the intramedullary nail through the distal locking screws.

The adjunctive screws were not used to fix each fracture line individually. Their purpose was to connect the distal articular fragments to the main fracture component, which was subsequently connected to the intramedullary nail through the distal locking screws, with the intramedullary nail then completing the overall fixation. This concept is related to the idea of overall connection described in previous calcaneal screw fixation studies (21, 22), but the specific screw configuration used for calcaneal fractures was not adopted, and an independent spatial construct of multiple adjunctive screws was not required. In this cohort, 83 of 98 patients received a single adjunctive screw. The approach therefore describes the fixation goal used in this cohort and does not represent a validated biomechanical configuration or a clinical decision-making system.

Preoperatively, the screw path was planned mainly according to the fracture-line distribution on CT, fragment size, and the anticipated spatial relationship between the adjunctive screws and the intramedullary nail. The number of screws was determined mainly according to fragment size, in combination with the specific fracture morphology, intraoperative fixation stability, and bone quality; no preset numerical criterion for screw number was used. During the study period, the basic fixation principles, the sequence of adjunctive screw and intramedullary nail insertion, and the implant system did not change substantially; the main change was increasing familiarity of the surgical team as cases accumulated.

### Surgical technique and implants

General anesthesia or neuraxial anesthesia was selected according to the fracture, the soft-tissue condition, and the anesthetic assessment. Patients were placed supine, with routine skin preparation and draping, and a tourniquet was used when necessary. For fractures requiring reduction, closed reduction or limited open reduction was performed, and Kirschner wires or reduction clamps were used for temporary fixation when necessary.

In all 98 patients, adjunctive screw fixation of the distal articular fragments was completed first, followed by reaming and insertion of the tibial intramedullary nail; no patient underwent intramedullary nail insertion before adjunctive fixation was completed (Figure 2). All intramedullary nails were inserted through an infrapatellar approach, mainly because the fractures in this cohort were located in the tibial shaft or the distal tibia and reduction of a proximal tibial fracture was not required. The tibial intramedullary nails and the associated adjunctive screws used during the study period were all manufactured by Tianjin Zhengtian Medical Instrument Co., Ltd. (Tianjin, China), and the implant system was not changed during the study period.

**Figure 2:**
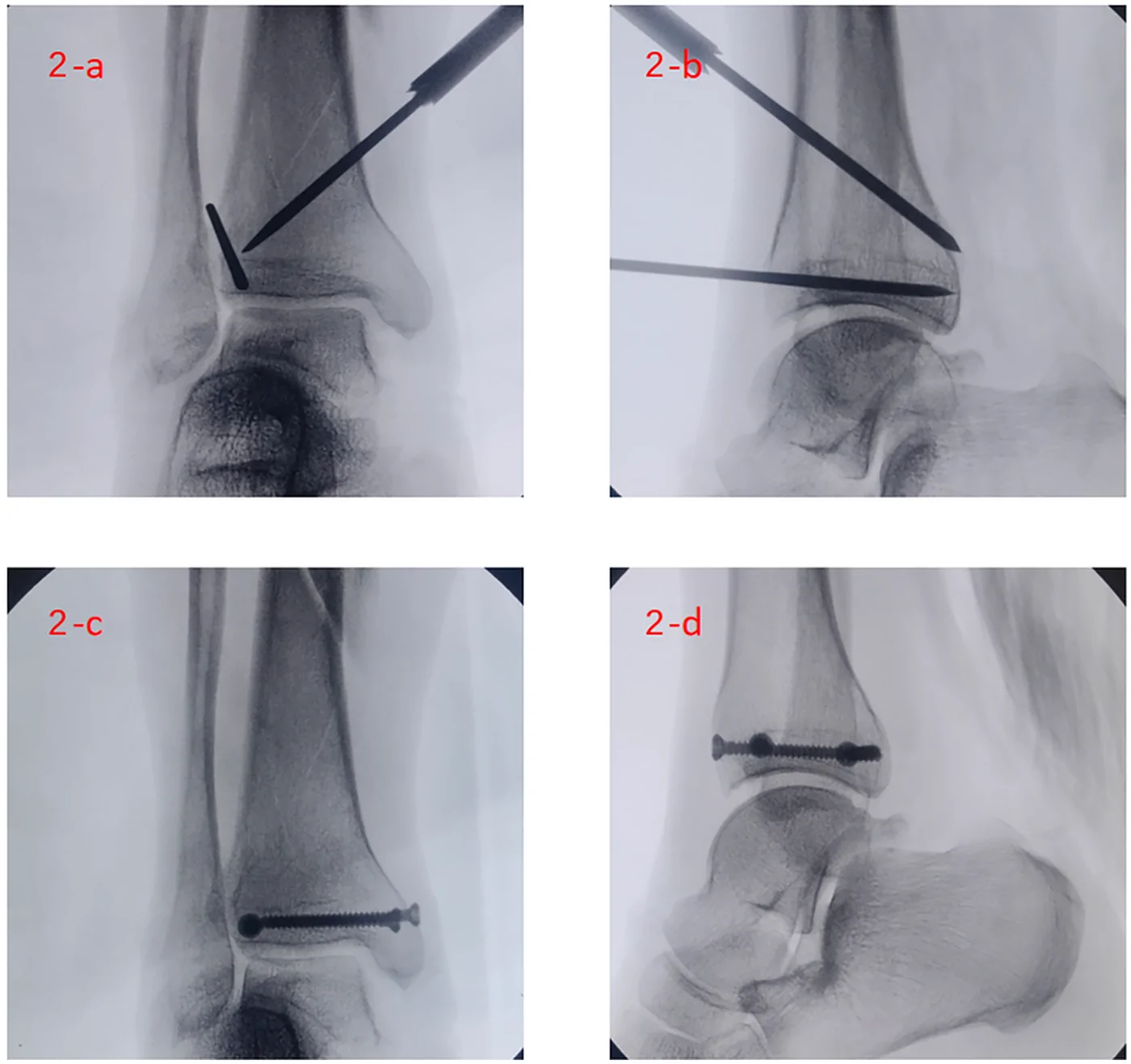
Representative intraoperative fluoroscopic images of adjunctive screw fixation of a distal tibial articular fracture line. **(a,b)** Anteroposterior and lateral views during establishment of the planned screw path across the target fracture line. **(c,d)** Anteroposterior and lateral views after definitive adjunctive screw placement. In all 98 patients in this cohort, the adjunctive screws were placed before reaming and intramedullary nail insertion; the sequence of adjunctive screw and intramedullary nail insertion was not individualized.

For relatively displaced distal articular fragments, 3.5-mm cannulated screws were used; for minimally displaced or occult articular fractures, 3.5-mm cortical screws were used. The specific screw type was determined by the surgeon according to the degree of displacement and the fixation requirement, and no uniform numerical displacement threshold was set. Lag screw technique was not used for adjunctive fixation in this cohort, and no blocking (Poller) screws were used.

Intraoperative C-arm fluoroscopy was used to assess fracture reduction, the position of the articular fragments, the position of the intramedullary nail, and screw length, with particular attention to the spatial relationships between the adjunctive screws and the distal end of the intramedullary nail and the distal locking screws. The specific screw corridor, direction, and number of screws were adjusted according to the individual fracture morphology, but the basic sequence of completing adjunctive fixation first and then reaming and inserting the intramedullary nail was not changed. After satisfactory reduction and implant positions were confirmed, the surgical field was irrigated and the incision was closed in layers.

Associated fibular fractures were managed according to the fracture location and the fixation requirement. Distal fibular fractures were treated with plate fixation; fractures of the distal fibular shaft were treated with plate fixation or intramedullary wire fixation; proximal and middle-to-upper shaft fractures were generally not directly fixed; and when an upper shaft fracture was combined with a distal fibular fracture, only the distal fracture was fixed. Fibular fracture location was recorded as the proximal fibula (head and neck), the upper, middle, or lower shaft, or the distal fibula, and fractures involving two levels were recorded as combined categories. Postoperative brace protection was provided when necessary. Syndesmotic stability was judged from the intraoperative clinical and imaging assessment. Because this was a retrospective study, the available records were insufficient to confirm whether all patients underwent a uniform syndesmotic stress test and to specify the method used.

### Retrospective classification of adjunctive screw corridors

According to the actual entry and exit points, the course, and the fixation target of the screws identified from operative records and postoperative imaging, the adjunctive screw paths used in this cohort were retrospectively summarized into four types: the tibiofibular incisura corridor, the anterior cortical corridor, the posterior cortical corridor, and the medial malleolar corridor (Figure 3). These names mainly describe the typical anatomical course and fixation target and do not represent standardized corridors with a uniform entry point, fixation angle, or screw length. The specific screw configuration was adjusted according to the individual osseous anatomy, the fracture-line course, fragment size, the reduction status, and the position of the distal end of the intramedullary nail and the distribution of the distal locking holes.

**Figure 3:**
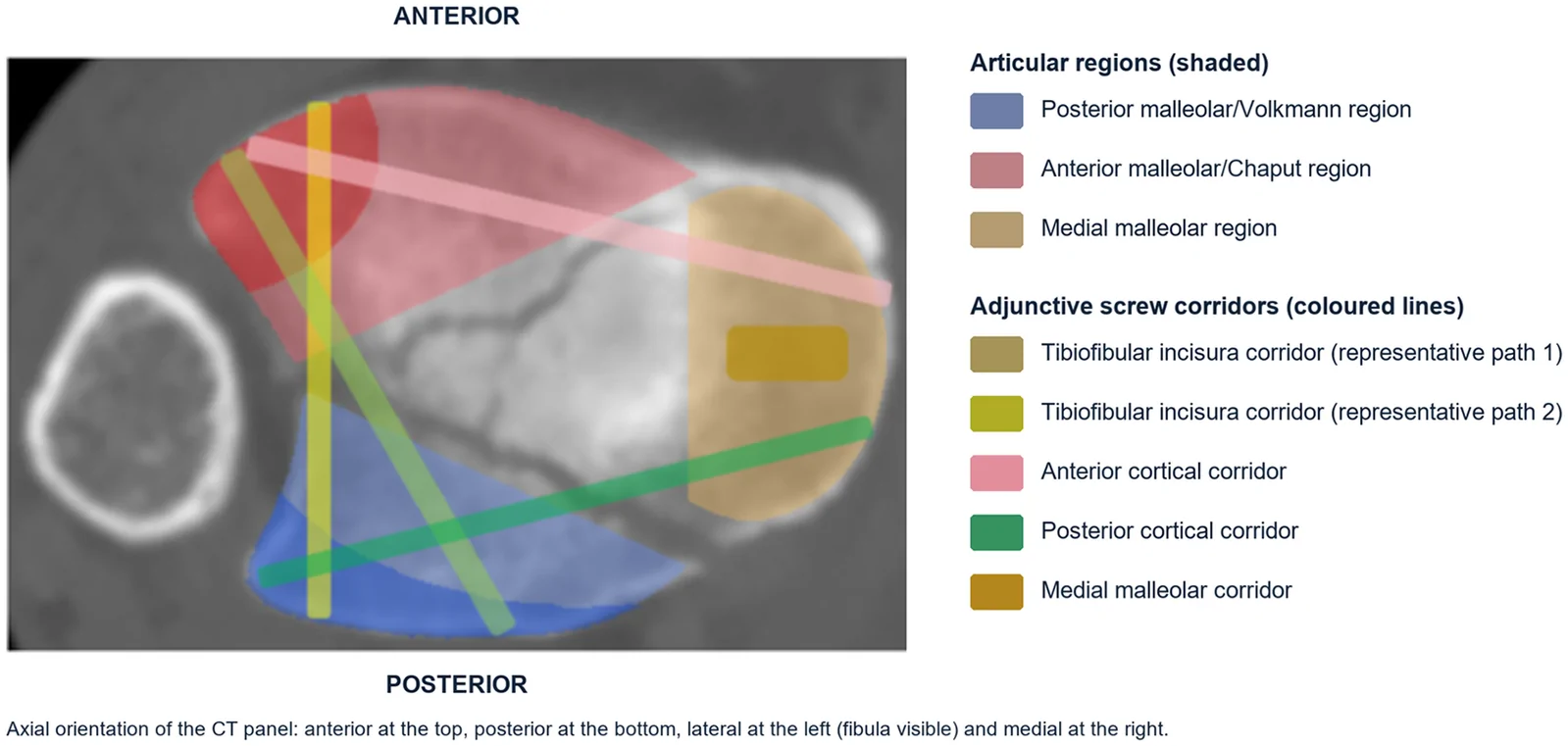
Schematic overview of the representative adjunctive screw corridors in the distal tibia, superimposed on an axial CT map of the distal tibia. The image is oriented with the anterior side at the top, the posterior side at the bottom, the lateral side at the left, where the fibula is visible, and the medial side at the right. The shaded areas indicate the articular regions: the posterior malleolar/Volkmann region (blue), the anterior malleolar/Chaput region (red), and the medial malleolar region (yellow-brown). The coloured lines indicate the representative corridors: two representative paths of the tibiofibular incisura corridor (olive), and the anterior cortical (pink), posterior cortical (green), and medial malleolar (dark yellow) corridors. The depicted paths indicate general fixation targets and anatomical directions rather than fixed, standardized entry points, insertion angles, or screw lengths.

#### Tibiofibular incisura corridor

When a screw entered from the anterolateral distal tibia or the region of the tibiofibular incisura and was directed posteriorly or posteromedially to connect and fix the anterior malleolar/Chaput fragment, the posterior malleolar/Volkmann fragment, or a continuous anterior-posterior fracture line, it was assigned to the tibiofibular incisura corridor (Figure 4). Use of this corridor did not require simultaneous involvement of the anterior and posterior regions, and in this cohort it was also used for isolated anterior or isolated posterior involvement. Anteroposterior and lateral fluoroscopic views of the ankle were routinely obtained intraoperatively, with an additional mortise view when necessary, to confirm that the screw had not entered the joint and to assess its spatial relationship with the distal end of the intramedullary nail and the distal locking screws.

**Figure 4:**
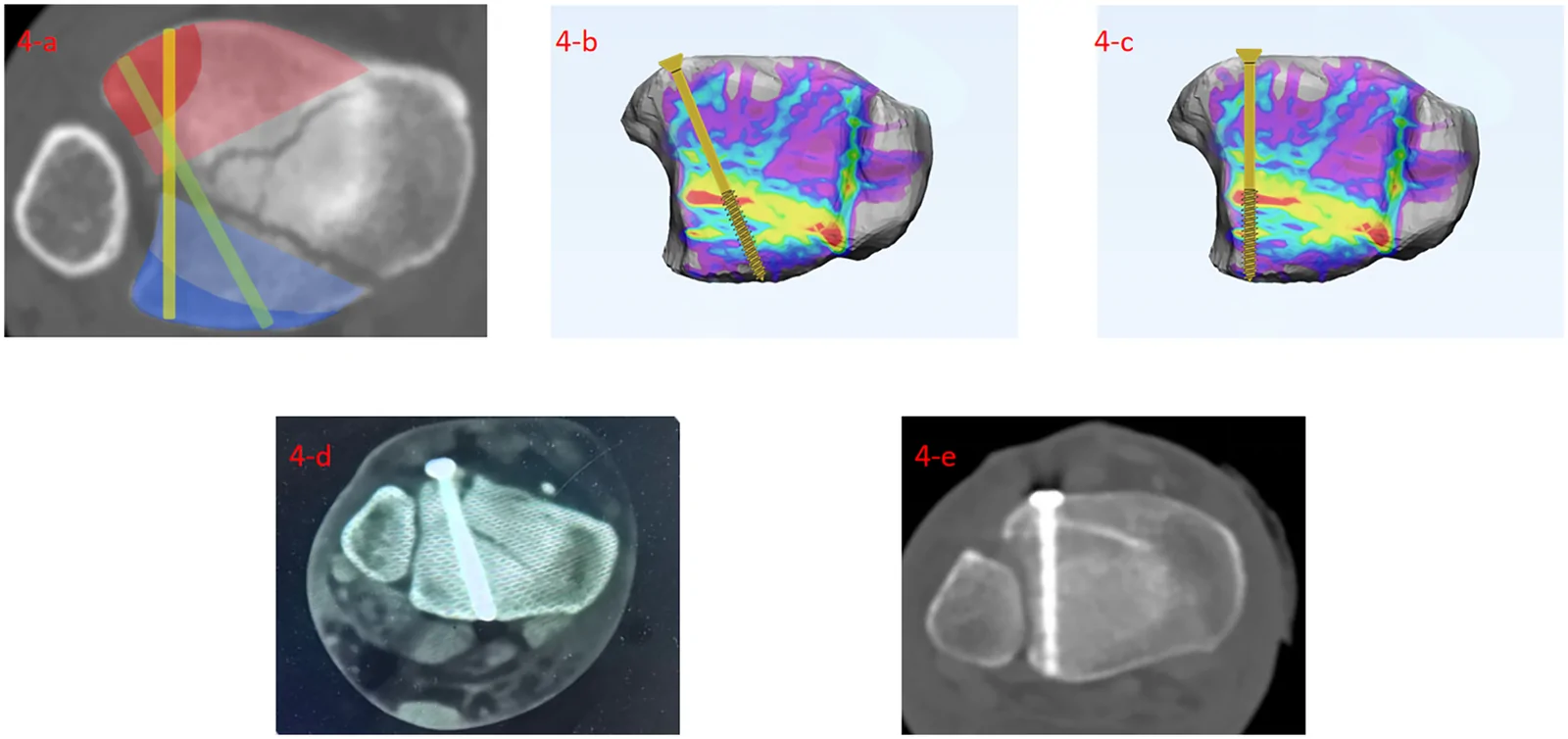
Tibiofibular incisura corridor. **(a)** Axial CT schematic showing two representative screw paths originating from the anterolateral distal tibia or the tibiofibular incisura and directed toward the posterior or posteromedial distal tibia. **(b,c)** Three-dimensional views of the two representative paths superimposed on the fracture-line density map. **(d,e)** Postoperative axial CT images showing screws placed along the corresponding paths. This corridor may be used to control anterior malleolar/Chaput, posterior malleolar/Volkmann, or continuous anterior-posterior fracture lines, and does not require simultaneous involvement of the anterior and posterior regions.

#### Anterior cortical corridor

The anterior cortical corridor was mainly used when a fracture line in the medial malleolar region extended toward the anterior malleolar/Chaput region and the medial and anterior fragments needed to be connected (Figure 5). The screw was usually inserted from the medial or anteromedial side toward the anterolateral side, following a course in the subcortical region of the anterior distal tibia. Adjacent tendons and neurovascular structures were considered during insertion, and fluoroscopy was used to confirm that the screw was located within the bone proximal to the articular surface.

**Figure 5:**
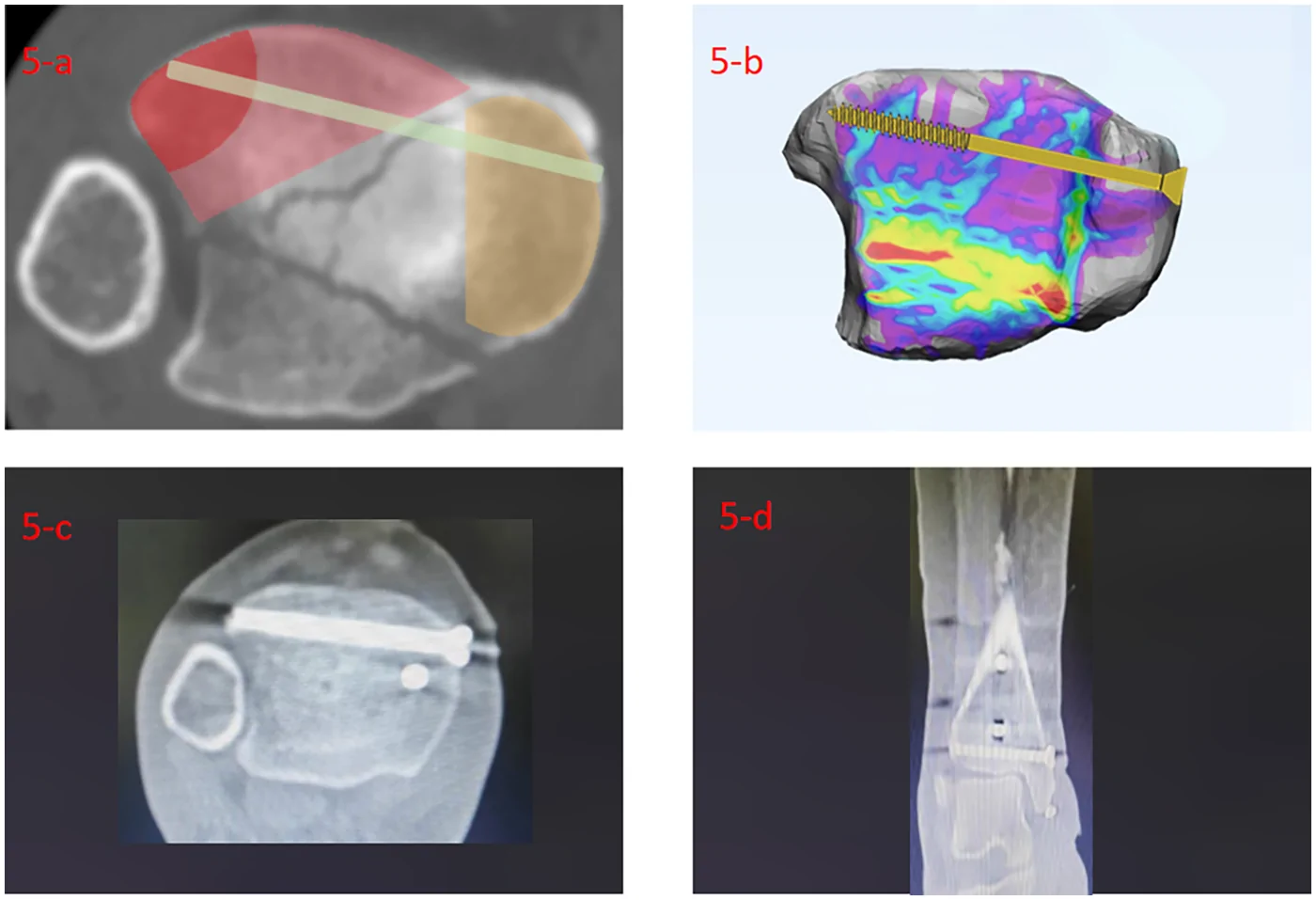
Anterior cortical corridor. **(a)** Axial CT schematic showing a path from the medial or anteromedial distal tibia toward the anterolateral side, close to the anterior cortex. **(b)** Three-dimensional view of the representative screw path relative to the fracture-line density map. **(c,d)** Postoperative axial and coronal CT images showing screw placement through the anterior cortical corridor. This corridor is mainly intended to connect and stabilize fracture lines extending from the medial malleolar region toward the anterior malleolar/Chaput region.

#### Posterior cortical corridor

The posterior cortical corridor was mainly used when a fracture line in the medial malleolar region extended toward the posterior malleolar/Volkmann region and the medial and posterior fragments needed to be connected (Figure 6). The screw was usually inserted from the medial side toward the posterolateral or posterior side, following a course in the subcortical region of the posterior distal tibia. Screw direction and length were controlled to avoid obvious penetration beyond the posterior cortex and irritation of adjacent tendons or neurovascular structures.

**Figure 6:**
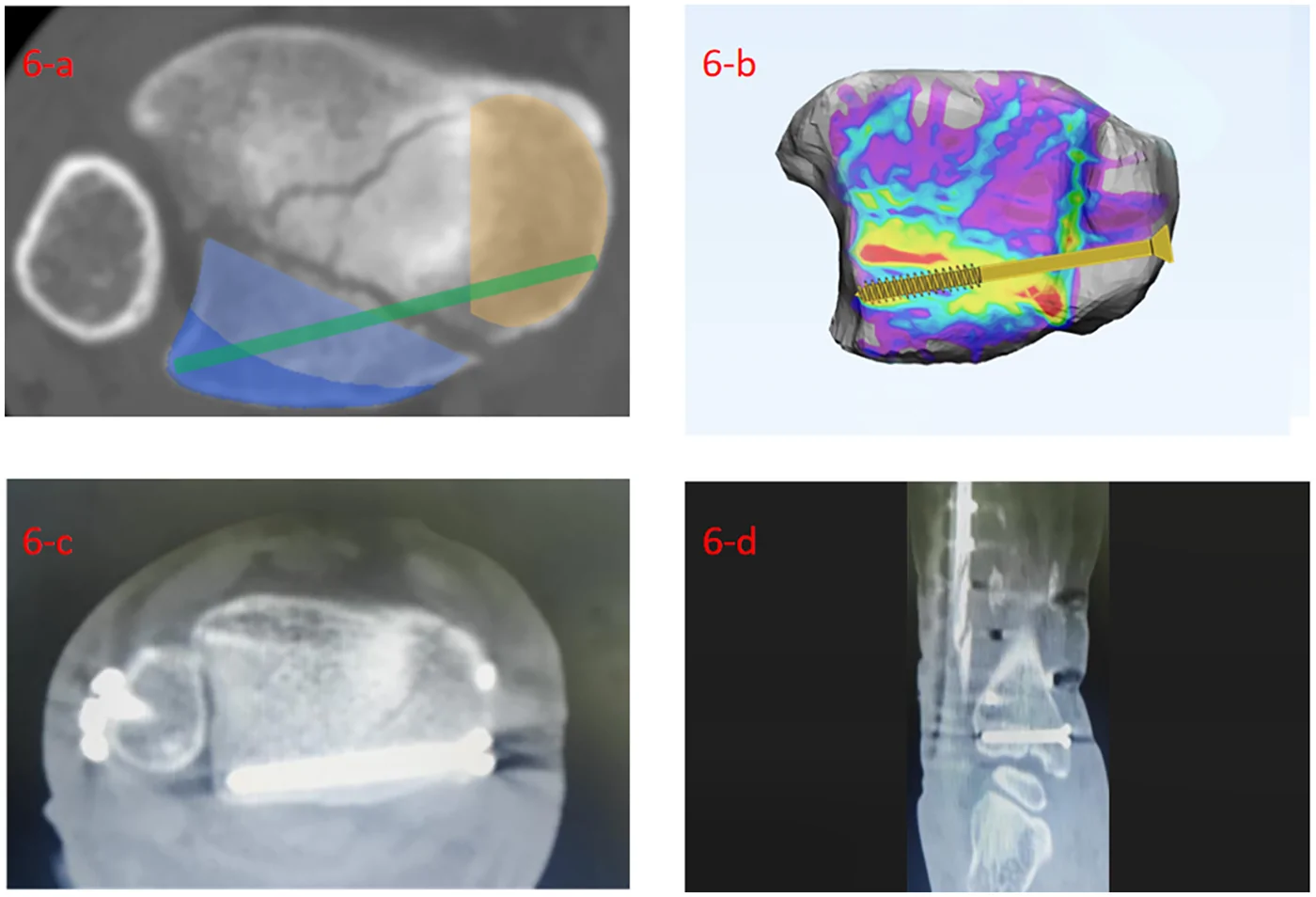
Posterior cortical corridor. **(a)** Axial CT schematic showing a path from the medial distal tibia toward the posterior or posterolateral side, close to the posterior cortex. **(b)** Three-dimensional view of the representative screw path relative to the fracture-line density map. **(c,d)** Postoperative axial and coronal CT images showing screw placement through the posterior cortical corridor. This corridor is mainly intended to connect and stabilize fracture lines extending from the medial malleolar region toward the posterior malleolar/Volkmann region.

#### Medial malleolar corridor

The medial malleolar corridor was mainly used to fix a separate medial malleolar fragment or a medial articular fracture line. The screw was inserted from the tip or the distal part of the medial malleolus toward the proximal tibia (Figure 7). In some patients, this corridor was combined with other adjunctive corridors, and the specific configuration was determined by the combination of involved articular regions and the overall fixation requirement.

**Figure 7:**
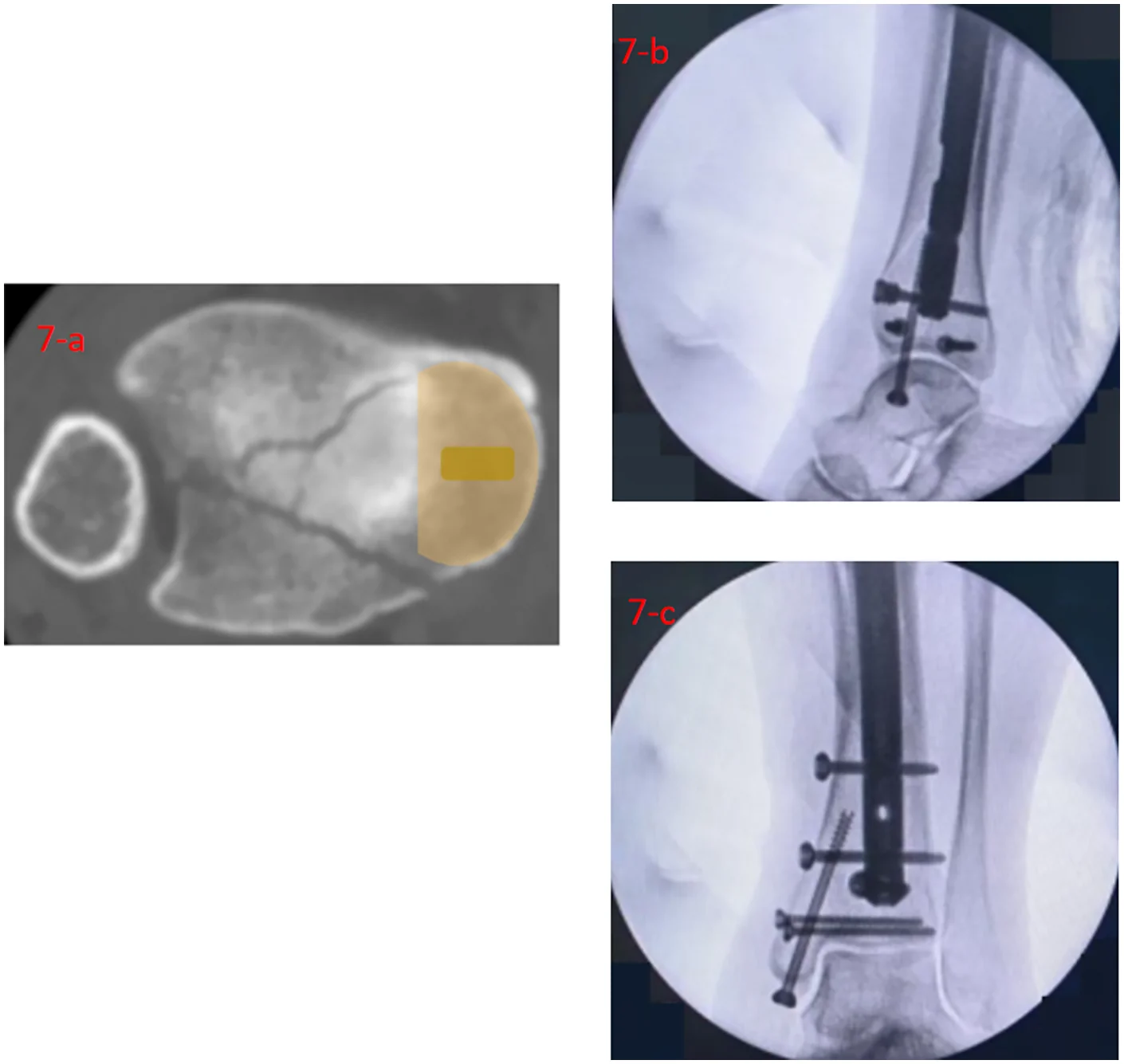
Medial malleolar corridor. **(a)** Axial CT schematic showing the medial malleolar region and a representative corridor for fixation of a medial malleolar fragment or a medial articular fracture line. **(b,c)** Intraoperative lateral and anteroposterior fluoroscopic views showing medial malleolar screw placement in combination with tibial intramedullary nailing. The corridor is directed from the tip or the distal part of the medial malleolus toward the proximal tibia and may be used alone or in combination with other adjunctive screw corridors.

### Postoperative management

Postoperative prophylactic antibiotics were given according to the perioperative management protocol and the condition of the incision. Early active and passive ankle motion was encouraged under brace protection, and full weight bearing was avoided in the early postoperative period. Weight-bearing progression was determined by the treating physician according to the fracture type, the stability of the articular fixation, callus formation, and follow-up imaging. After imaging showed a trend toward fracture healing and clinical assessment confirmed stable fixation, patients gradually progressed to full weight bearing.

### Data collection and outcome assessment

Age, sex, injury mechanism, fracture side, the AO/OTA code of the main tibial fracture, and the involved regions of the distal tibial articular surface were collected. Perioperative data included operative time, intraoperative blood loss, the adjunctive screw corridors actually used, and the number of screws; intraoperative neurovascular injury and obvious spatial interference between the adjunctive screws and the intramedullary nail or the distal locking screws were also recorded. Follow-up variables included time to full weight bearing, articular redisplacement, fixation failure, infection, wound complications, and secondary surgery.

Time to full weight bearing was defined as the interval from the day of surgery to the outpatient follow-up visit at which the patient was confirmed to be able to stand and walk independently without crutches. This variable therefore reflects the time confirmed at an outpatient visit rather than the exact date of first achieving full weight-bearing capacity determined by continuous daily assessment. At the final follow-up, ankle function was assessed with the AOFAS ankle-hindfoot score, and pain was assessed with the VAS.

Active ankle dorsiflexion was measured with a standard goniometer. The patient was seated with the examined lower limb placed on the examination table and the knee extended. The axis of the goniometer was placed over the lateral malleolus, the stationary arm was aligned with the longitudinal axis of the leg, and the moving arm was aligned with the longitudinal axis of the fifth metatarsal; the active dorsiflexion angle was recorded. The affected and contralateral sides were measured under the same conditions, and the difference between the two sides was calculated.

All patients underwent CT on the first postoperative day to assess the reduction of the articular fragments and the position of the implants. Subsequent follow-up used conventional radiographs to assess fracture healing, the position of the articular fragments, and the status of the internal fixation. Articular redisplacement was defined as new or increased fragment displacement or articular incongruity on follow-up imaging compared with the early postoperative images. Fixation failure was defined as breakage, loosening, or migration of the intramedullary nail, the locking screws, or the adjunctive screws during follow-up. Both imaging outcomes were assessed qualitatively, and no uniform numerical threshold in millimeters was predefined.

Perioperative variables and complications were analyzed in all 98 patients. One patient underwent further treatment for infection at the tibial shaft fracture site, did not complete the scheduled final functional and ankle range-of-motion assessment, and was nevertheless included in the complication analysis. Time to full weight bearing, the AOFAS score, the VAS score, and the difference in ankle dorsiflexion angle were analyzed in the remaining 97 patients.

### Statistical analysis

All data were analyzed using SPSS version 26.0, and the analysis was primarily descriptive. Continuous variables were tested for normality; normally distributed variables are presented as mean ± standard deviation, and non-normally distributed variables as median and interquartile range. Categorical variables are presented as counts and percentages.

The same patient could have involvement of multiple distal articular regions and could receive adjunctive screws through multiple corridors; the corresponding categories were therefore counted separately according to actual involvement or use, and the percentages across categories could sum to more than 100%. In Table 3, the within-group proportion of use of each corridor was calculated using the number of patients with the corresponding involvement pattern as the denominator. Because the corridors were not mutually exclusive patient groups and some corridors were used in only a small number of patients, no comparison of the relative merits of different corridors and no inferential test of the association between involvement pattern and corridor were performed.

Interobserver agreement was assessed with Cohen's kappa and the agreement rate, and the standard error and 95% confidence interval of kappa were reported.

## Results

### Patient characteristics

Of the 110 patients treated with this combined fixation during the study period, 98 were included in the analysis and 12 were excluded because of a follow-up duration shorter than 12 months or incomplete imaging records. The 98 included patients comprised 62 men and 36 women, aged 19–80 years, with a mean age of 45.67 ± 13.61 years. Forty-two fractures involved the left side and 56 the right side. The AO/OTA codes of the main tibial fracture were 42A1 in 54 patients, 42B1 in 23, 42C1 in 8, and 43A1 in 13. Of the 95 patients with an associated fibular fracture, 55 underwent no direct fibular fixation, 34 underwent plate fixation, and 6 underwent intramedullary wire fixation. The fibular fracture involved the distal fibula in 23 patients, the proximal fibula in 21, the upper shaft in 17, the middle shaft in 15, the lower shaft in 13, the proximal fibula together with the middle shaft in 2, and the upper shaft together with the distal fibula in 4 (Table 1). Based on the available medical records and operative notes, no definite syndesmotic separation was identified in this cohort, and no patient underwent syndesmotic screw fixation. Injury mechanisms included a slip and fall in 59 patients, an electric bicycle fall in 16, a fall down stairs in 8, a road traffic injury in 8, a bicycle fall in 4, a crush injury from a falling heavy object in 2, and a fall from height in 1 (Table 1).

### Distribution of distal articular involvement

All 98 patients had involvement of the distal tibial articular surface confirmed by preoperative CT. The posterior malleolar/Volkmann region was involved in 76 patients (77.6%), the anterior malleolar/Chaput region in 40 (40.8%), and the medial malleolar region in 23 (23.5%) (Table 2a). 64 patients had single region involvement, including isolated posterior malleolar/Volkmann involvement in 48, isolated anterior malleolar/Chaput involvement in 11, and isolated medial malleolar involvement in 5. 27 patients had involvement of two regions, including posterior plus anterior involvement in 16, posterior plus medial involvement in 5, and anterior plus medial involvement in 6. A further 7 patients had involvement of all three regions (Table 2b).

**Table 2b:** Combination patterns of involved regions.

Involvement pattern	n	Percentage
Posterior malleolar/Volkmann region only	48	49.0%
Anterior malleolar/Chaput region only	11	11.2%
Medial malleolar region only	5	5.1%
Posterior malleolar + anterior malleolar regions	16	16.3%
Posterior malleolar + medial malleolar regions	5	5.1%
Anterior malleolar + medial malleolar regions	6	6.1%
Posterior malleolar + anterior malleolar + medial malleolar regions	7	7.1%
Total	98	100.0%

Single-region involvement, two-region involvement, and three-region involvement were present in 64 (65.3%), 27 (27.6%), and 7 (7.1%) patients, respectively.

### Interobserver agreement

The agreement rates of the two observers for involvement of the posterior malleolar/Volkmann, anterior malleolar/Chaput, and medial malleolar regions were 96.9%, 98.0%, and 98.0%, respectively, with corresponding Cohen's kappa values of 0.924 (95% CI 0.840-1.000), 0.957 (95% CI 0.899-1.000), and 0.939 (95% CI 0.856–1.000). For combinations of involved regions, the agreement rate was 95.9% and the kappa value was 0.943 (95% CI 0.888–0.998). Complete results of the agreement analysis are shown in Supplementary Table S1.

### Corridor application and number of screws

The adjunctive screw corridors actually used in each articular involvement pattern are shown in Table 3. All 48 patients with isolated posterior malleolar/Volkmann involvement and all 11 patients with isolated anterior malleolar/Chaput involvement used the tibiofibular incisura corridor. Among the 5 patients with isolated medial malleolar involvement, the anterior cortical corridor and the posterior cortical corridor were each used in 4 patients. Overall, the anterior cortical, posterior cortical, and medial malleolar corridors were mainly used in patients with medial malleolar involvement or with combined involvement of multiple articular regions.

**Table 3:** Distribution of adjunctive screw corridors according to patterns of articular involvement.

Involvement pattern	n	Tibiofibular incisura corridor	Anterior cortical corridor	Posterior cortical corridor	Medial malleolar corridor	Single/multiple corridors
Posterior malleolar/Volkmann region only	48	48 (100.0%)	0 (0.0%)	0 (0.0%)	0 (0.0%)	48/0
Anterior malleolar/Chaput region only	11	11 (100.0%)	0 (0.0%)	0 (0.0%)	0 (0.0%)	11/0
Medial malleolar region only	5	1 (20.0%)	4 (80.0%)	4 (80.0%)	1 (20.0%)	1/4
Posterior malleolar + anterior malleolar regions	16	16 (100.0%)	1 (6.3%)	1 (6.3%)	0 (0.0%)	15/1
Posterior malleolar + medial malleolar regions	5	5 (100.0%)	0 (0.0%)	0 (0.0%)	1 (20.0%)	4/1
Anterior malleolar + medial malleolar regions	6	5 (83.3%)	2 (33.3%)	1 (16.7%)	4 (66.7%)	2/4
Posterior malleolar + anterior malleolar + medial malleolar regions	7	7 (100.0%)	1 (14.3%)	1 (14.3%)	0 (0.0%)	6/1
Total	98	93 (94.9%)	8 (8.2%)	7 (7.1%)	6 (6.1%)	87/11

A patient could receive adjunctive screws through more than one corridor; therefore, the sum across corridor categories may exceed the total number of patients. Percentages in the corridor columns were calculated using the number of patients with the corresponding involvement pattern as the denominator. The single/multiple corridor column reflects the number of corridor types used, not the number of screws.

Overall, the tibiofibular incisura corridor was used in 93 of 98 patients (94.9%), the anterior cortical corridor in 8 (8.2%), the posterior cortical corridor in 7 (7.1%), and the medial malleolar corridor in 6 (6.1%). Eighty-seven patients (88.8%) used only one corridor, and 11 (11.2%) used two or more corridors. The number of adjunctive screws ranged from 1 to 4, with a mean of 1.23 ± 0.62. One, two, three, and four adjunctive screws were used in 83, 9, 4, and 2 patients, respectively (Table 4).

**Table 4:** Application of adjunctive screw corridors and number of screws.

Variable	Cases or value	Percentage
Tibiofibular incisura corridor	93	94.9%
Anterior cortical corridor	8	8.2%
Posterior cortical corridor	7	7.1%
Medial malleolar corridor	6	6.1%
Single-corridor fixation	87	88.8%
Multiple-corridor fixation	11	11.2%
Number of adjunctive screws	1.23 ± 0.62	-
1 adjunctive screw	83	84.7%
2 adjunctive screws	9	9.2%
3 adjunctive screws	4	4.1%
4 adjunctive screws	2	2.0%

Corridor categories were not mutually exclusive. A patient could receive adjunctive screws through more than one corridor; therefore, the sum of percentages across corridor categories may exceed 100%.

### Perioperative, follow-up, and functional outcomes

Mean operative time was 73.45 ± 7.88 min, mean intraoperative blood loss was 67.56 ± 10.46 mL, and mean follow-up duration was 16.13 ± 2.20 months. Among the 97 patients who completed the relevant outcome assessments, the mean time to full weight bearing was 64.81 ± 16.91 days. At the final follow-up, the mean AOFAS ankle-hindfoot score was 91.67 ± 3.13, the mean VAS pain score was 1.36 ± 0.50, and the mean difference in ankle dorsiflexion angle between the affected and contralateral sides was 6.06 ± 3.27 degrees (Table 5).

**Table 5:** Perioperative, follow-up, functional, and complication outcomes.

**Variable**	**Value**
Operative time, min	73.45 ± 7.88
Intraoperative blood loss, mL	67.56 ± 10.46
Follow-up duration, months	16.13 ± 2.20
Time to full weight bearing, days	64.81 ± 16.91
AOFAS ankle-hindfoot score	91.67 ± 3.13
VAS pain score	1.36 ± 0.50
Difference in ankle dorsiflexion, degrees	6.06 ± 3.27
Delayed healing of the anterior ankle incision used for distal locking screws	2 cases (2.0%)
Infection at the tibial shaft fracture site	1 case (1.0%)
Intraoperative neurovascular injury	0
Obvious spatial interference between the adjunctive screws and the nail or the distal locking screws	0
Obvious articular redisplacement on available follow-up imaging	0 observed
Fixation failure	0 observed

Perioperative and complication outcomes were analyzed in the 98 patients with complete follow-up; the 12 patients excluded because of insufficient follow-up or incomplete imaging were not included in these analyses. One patient underwent further treatment for postoperative infection and did not complete the scheduled final functional and ankle range-of-motion assessment; therefore, time to full weight bearing, the AOFAS score, the VAS score, and the difference in ankle dorsiflexion angle were analyzed in 97 patients. Articular redisplacement and fixation failure were assessed qualitatively on the available follow-up images; the values indicate that no obvious abnormality was observed and do not exclude subtle changes. AOFAS, American Orthopaedic Foot & Ankle Society; VAS, visual analog scale.

### Complications

All 98 patients with complete follow-up were included in the complication analysis; the 12 patients excluded for insufficient follow-up or incomplete imaging were not included.]

Based on the available medical records and follow-up data, no intraoperative neurovascular injury and no obvious spatial interference between the adjunctive screws and the intramedullary nail or the distal locking screws were recorded. All 98 patients underwent CT on the first postoperative day. During follow-up, fracture reduction and the status of the internal fixation were assessed with conventional radiographs, and no obvious articular redisplacement or fixation failure was observed among these 98 patients (Table 5).

## Discussion

This study retrospectively summarized the clinical data of 98 patients with spiral tibial fractures involving the distal tibial articular surface who were treated with intramedullary nailing combined with adjunctive screw fixation, building on our previous CT-based morphological study. According to the actual course and fixation target of the adjunctive screws, the screw paths used in this cohort were summarized into four corridors: the tibiofibular incisura, anterior cortical, posterior cortical, and medial malleolar corridors. The corridors actually used showed a certain distribution across different patterns of articular involvement. At the same time, the adjunctive screws did not act independently of the intramedullary nail; by connecting the distal articular fragments to the main fracture component, they together with the intramedullary nail and the distal locking screws formed the overall fixation. Compared with previous studies that focused mainly on fracture healing, alignment, and functional outcomes (14–19), this study provides further technical information on the actual course of the adjunctive screws, their fixation targets, and, in representative cases, their relationship with the intramedullary nail system.

Previous studies have reported the clinical use of intramedullary nailing combined with adjunctive screws in selected distal tibial fractures with simple articular extension (14–19). Overall, the previous literature has focused more on the clinical feasibility of combined fixation, fracture healing, and postoperative function, whereas the way in which adjunctive screws are arranged around different fragments and coordinated with the distal end of the intramedullary nail and the locking screws has been reported relatively rarely. This study therefore extends the focus to the configuration and screw paths of the adjunctive fixation itself.

This technical question is closely related to the morphology of spiral tibial fractures involving the distal tibial articular surface. Our previous CT study showed that, in addition to the posterior malleolar/Volkmann region, which has traditionally received more attention, these fractures may also involve the anterior malleolar/Chaput region and the medial malleolar region, and some fracture lines may extend continuously across two or even three regions (20). Distal articular injury therefore does not always present as an isolated posterior malleolar fragment. When a fracture line extends continuously anteriorly, medially, or across multiple regions, the connection between the fragments that need to be addressed also becomes more complex. In terms of the screws actually placed in this cohort, the tibiofibular incisura corridor could be used for anterior, posterior, and continuous anterior-posterior fracture lines, whereas the anterior cortical, posterior cortical, and medial malleolar corridors were more often used in patients with medial malleolar involvement or combined involvement of multiple regions. These distributions reflect differences in fixation target and screw direction among different fracture morphologies, but they do not mean that a given fracture morphology can only correspond to one specific corridor.

Based on these characteristics, the screws in this cohort were placed according to the spatial relationships between the fragments rather than as a separate screw for each individual fracture line, and the overall fixation was completed with the intramedullary nail. That 83 of the 98 patients received only one adjunctive screw also indicates that this approach did not require multiple screws to form a complex three-dimensional structure; the emphasis was on the connecting role of the adjunctive screws within the overall fixation construct. The concept has some similarity to the overall connection concept reflected in previous calcaneal fixation studies (21, 22), but the distal tibia and the calcaneus differ markedly in anatomy and loading environment, and the borrowing therefore lies mainly in the fixation concept rather than in a specific configuration or mechanical performance.

In addition to fracture morphology, spatial coordination between implants is a key issue in the design of adjunctive fixation. Previous studies have analyzed the distal position, endpoint, alignment, and osseous corridor of the tibial intramedullary nail from different perspectives (23–26). In fractures involving the articular surface, the adjunctive screws are usually located proximal to the articular surface, whereas the distal end of the intramedullary nail and the distal locking screws also occupy the limited osseous space of the distal tibia. The adjunctive screws therefore need both to cross or control the target fracture line and to avoid interfering with the insertion of the intramedullary nail and the locking screws. For this reason, the term “corridor” in this article is better understood as a type of screw path with a similar fixation target and anatomical direction rather than a fixed entry point, insertion angle, or screw length. In practice, the specific configuration still needs to be adjusted according to the individual CT morphology, the fracture reduction, and the intraoperative fluoroscopic findings.

The sequence of fixation is another important aspect of combined fixation. All 98 patients in this cohort completed adjunctive fixation of the distal articular fragments before reaming and intramedullary nail insertion. For fragments that were already displaced, reduction and fixation first helped to maintain their relative position with respect to the main fracture component; for fragments without displacement or with minimal displacement, adjunctive fixation was used mainly to reduce the possibility of secondary displacement during reaming and nail insertion. The study by Kempegowda et al. on tibial shaft fractures with posterior malleolar fractures indicated that the sequence of fixation may affect the position of the posterior malleolar fragment during nail insertion (13). The treatment principle in this cohort is consistent with that idea, but the cases also included anterior malleolar, medial malleolar, and multi-region involvement, so the scope of application is more complex than that of an isolated posterior malleolar fracture. For articular injuries of different degrees and at different sites, the specific indications for adjunctive fixation still require further study.

Bony fixation and the safety of the surrounding soft tissues should also be considered during screw insertion. Multiple tendons and important neurovascular structures surround the distal tibia, and different screw directions involve different anatomical risks. Previous studies have investigated the neurovascular structures anterior to the ankle, the syndesmotic screw insertion region, the posterolateral soft-tissue structures, and the safe zone for medial malleolar screws (27–31), and these studies provide a reference for understanding the local anatomical relationships of the different screw directions in this article. No intraoperative neurovascular injury related to the adjunctive screws was recorded in this cohort, but systematic anatomical measurement of these four corridors is still lacking. In addition, local bone quality and cortical support may affect screw fixation, and the mechanical behavior of the different corridors within the overall fixation construct remains to be further verified.

At the final follow-up, the 97 patients who completed the relevant assessments had a mean AOFAS ankle-hindfoot score of 91.67 ± 3.13, a mean VAS pain score of 1.36 ± 0.50, and a mean difference in ankle dorsiflexion angle between the affected and contralateral sides of 6.06 ± 3.27 degrees. These results reflect the function, pain, and ankle mobility of the patients in this cohort after combined fixation, but dorsiflexion on the affected side was still reduced to some extent, indicating that functional recovery does not equate to complete recovery of ankle range of motion. The reasons may be related to the initial articular injury, the condition of the soft tissues, postoperative immobilization, and the rehabilitation process. Regarding complications, two patients developed delayed healing of the anterior ankle incision used for distal locking screws, and one patient developed an infection at the tibial shaft fracture site and underwent further surgery; no obvious articular redisplacement or fixation failure was observed on the available follow-up images. These results describe the clinical course after combined fixation in this cohort, but because there was no concurrent control group receiving another fixation method, the functional and imaging results cannot be attributed to a particular screw corridor or to adjunctive fixation alone.

This study has several limitations. It was a single-center retrospective case series without a control group, and the cohort was conditioned on receipt of the combined fixation, so the reported corridor frequencies describe this selected group and should not be interpreted as representing all spiral tibial fractures with distal articular extension. The specific screw plan was determined by a single surgical team and a single senior surgeon, so the four corridors may partly reflect one team's practice and should not be considered exhaustive. The relationship between the adjunctive screws and the intramedullary nail was illustrated with representative images rather than analyzed systematically. Inclusion required at least 12 months of follow-up with complete imaging records, and 12 of the 110 patients treated with this combined fixation were excluded for insufficient follow-up, so selection bias cannot be excluded and the absence of articular redisplacement and fixation failure applies only to the 98 patients with complete follow-up. In addition, 89 of the 98 patients overlapped with our previous morphological study, and the imaging and functional assessments were qualitative or unblinded, without anatomical or biomechanical validation. Future studies using three-dimensional CT, anatomical and biomechanical analysis, and prospective designs are needed to clarify the anatomical and mechanical characteristics of these corridors and their clinical value.

## Conclusion

This retrospective case series summarized the tibiofibular incisura, anterior cortical, posterior cortical, and medial malleolar corridors actually used when spiral tibial fractures involving the distal tibial articular surface were treated with intramedullary nailing combined with adjunctive screw fixation, and described the fixation target of each corridor. The adjunctive screws were used to connect the distal articular fragments to the main fracture component and then to complete the overall fixation together with the intramedullary nail; representative images illustrate, but do not systematically analyze, the relationship between the adjunctive screws and the intramedullary nail. These results provide descriptive information on the adjunctive fixation technique used in this cohort, and their value for guiding preoperative planning and surgical decision-making requires further validation.

## Data Availability

The raw data supporting the conclusions of this article will be made available by the authors, without undue reservation.
