# Supplementary material for: Adjunctive screw fixation and screw corridors for spiral tibial fractures involving the distal tibial articular surface: a retrospective case series
Source: Front Surg. 2026 Oct 22;13:1944619. doi: 10.3389/fsurg.2026.1944619 (PMC13737581; doi:10.3389/fsurg.2026.1944619)
Supplement: Supplementary file 1 [file Table1.docx]

**Supplementary Table S1**

Interobserver agreement for distal tibial articular involvement

Manuscript: Framework-Based Adjunctive Screw Fixation and Screw Corridors for Spiral Tibial Fractures Involving the Distal Tibial Articular Surface: A Retrospective Case Series

Supplementary Table S1. Interobserver agreement for distal tibial articular involvement

| **Assessment item** | **Agreement rate** | **Cohen's kappa** | **Standard error** | **95% CI** | **Agreement level** |
| --- | --- | --- | --- | --- | --- |
| Posterior malleolar/Volkmann region involvement | 96.9% | 0.924 | 0.043 | 0.840-1.000 | Almost perfect |
| Anterior malleolar/Chaput region involvement | 98.0% | 0.957 | 0.030 | 0.899-1.000 | Almost perfect |
| Medial malleolar region involvement | 98.0% | 0.939 | 0.043 | 0.856-1.000 | Almost perfect |
| Combination pattern of involved regions | 95.9% | 0.943 | 0.028 | 0.888-0.998 | Almost perfect |

*Note: Cohen's kappa, the agreement rate, and the 95% confidence interval of kappa were calculated from the original independent assessments of the two observers obtained before the consensus discussion. According to the Landis-Koch criteria, kappa values of 0.81-1.00 indicate almost perfect agreement. The 95% confidence intervals refer to the kappa estimates, not to the agreement rates.*

Abbreviation: CI, confidence interval.
